# Knowledge of Cervical Cancer and Human Papillomavirus Vaccines among Child-Bearing Aged Women in Hanoi, Vietnam

**DOI:** 10.31557/APJCP.2020.21.7.1951

**Published:** 2020-07

**Authors:** Nguyen Thi Ngoc Phuong, Le Thi Thanh Xuan, Le Thi Huong, Do Thi Thanh Toan, Jin Kyung Oh, Young Joo Won, Kui Son Choi

**Affiliations:** 1 *Graduate School of Cancer Science and Policy, National Cancer Center, Goyang, Gyeonggi-do, 10408, Republic of Korea. *; 2 *Institute for Preventive Medicine and Public Health, Hanoi Medical University, Hanoi, 10000, Vietnam. *; 3 *Cancer Registration and Statistic Branch, National Cancer Center, Goyang, Gyeonggi-do, 10408, Republic of Korea. *

**Keywords:** Cervical cancer, HPV vaccines, knowledge, Vietnam

## Abstract

**Objectives:**

The study sought to examine knowledge of cervical cancer and human papillomavirus (HPV) vaccines among child-bearing aged women in Hanoi, Vietnam**. **

**Methods:**

In 2016, 807 women aged 18 to 49 years were recruited from one urban area and one rural area in 2016 and were examined through face-to-face paper-based interviews. Pearson’s chi-square test and an independent t-test were utilized to compare awareness of cervical cancer and HPV vaccination among women according residential status.

**Results:**

Overall, 83.8% and 71.3% women had heard about cervical cancer and HPV vaccination, respectively. Mean knowledge scores for cervical cancer and HPV vaccination were 4.60±1.43 out of 7 and 1.53±1.35 out of 5, respectively. Women living in an urban area were more likely to be aware of cervical cancer and to be more knowledgeable of HPV vaccination than women in a rural area.

**Conclusions:**

Despite strong awareness, we found knowledge on cervical cancer and HPV vaccination to be alarmingly insufficient among Vietnamese women.

## Introduction

Cervical cancer poses a tremendous global health challenge, especially in less developed parts of the world where cervical cancer incidence and mortality rates can reach up to 80% (Bray et al.; World Health Organization, 2016). Safe sex behavior, getting an HPV vaccine, and cervical cancer screening are well-recognized ways to successfully prevent this cancer and lower mortality rates (World Health Organization, 2016). Since its launch in 2006, HPV vaccination has garnered consideration as an important life-saving intervention for millions of women throughout the world. Indeed, in the most developed parts of the world, where cervical cancer screening and HPV vaccination are readily available, cervical cancer incidence rates have dropped dramatically (Vaccarella et al., 2013). 

In Vietnam, cervical cancer ranks as the second most common cancer among women of ages ranging from 15 to 44 years (Bruni, 2017). To date, cervical cancer screening to detect cancer early is only offered in opportunistic screening programs in Vietnam, and few women elect to undergo cervical cancer screening using their own financial resources. Also, according to a report in 2016 by the United Nations, condom usage among men in Vietnam was extremely low, at less than 12% (United Nations, 2016). To prevent cervical cancer in Vietnam, a pilot program for HPV vaccination was implemented in 2008; however, until now, it has not been incorporated into the national immunization program. Moreover, with limited resources dedicated to the prevention of cervical cancer, raising awareness of cervical cancer and HPV vaccination has proven to be a major challenge (Bingham et al., 2009; Tung et al., 2016). 

The aim of this study was to examine knowledge of cervical cancer and HPV vaccination among women of child-bearing age in Hanoi, Vietnam using data collected in 2016. Understanding these data will help with designing and implementing successful vaccination programs through which to propel acceptance of HPV vaccination forward in Vietnamese society and minimizing the burden of this disease.

## Materials and Methods

*Data sources and Setting*

Data were obtained from a project funded by Pfizer, an American pharmaceutical company, entitled “Vaccination accessibility for child-bearing age women in low- and middle-income countries in Southeast Asia period 2016-2018,” which was conducted as a multi-country, pre- and post-evaluation, and community-based randomized control trial for improving awareness and utilization of several vaccine types (e.g., rubella, influenza, tetanus, hepatitis B, and HPV) among reproductive-aged women. In the current study, data collected for pre-evaluation were utilized.

*Study design and sample*

The present study was conducted as an observational cross-sectional study with a convenient multistage cluster sampling method. First, two districts, representative of residence status, were randomly chosen: Dong Da (urban area) and Ba Vi (rural area). Second, two health communes were randomly selected for these two areas. Finally, lists of all women who were pregnant or had just given birth in the last 12 months at the time of the survey were received from the local health centers.

The potential participants were recruited in this study if they met the following inclusion criteria: (1) women aged 18 to 49 years at the time of the survey; (2) currently living in the selected communes for at least 1 year and presence during the recruitment times; (3) pregnant or gave birth within the last 12 months (from April 16, 2015 to April 15, 2016); and (4) agreed to participate in this project.

Regarding sample size calculation, since no study has measured coverage of HPV vaccination among reproductive-aged women in Vietnam, we set P to equal 0.5. With 95% confidence intervals, a maximum permissible error (Δ) of 0.05 and a 10% drop out in case an individual refused to participate or was absent during the collection period, final sample sizes of 400 women in each district and 800 in total were calculated. Participants were then selected from two communal health units in Dong Da and Ba Vi from lists of 975 and 470 eligible women, respectively. A total of 807 women finally participated in this study.

*Study instrument*

Face-to-face interviews were conducted by a professional researcher using a structured questionnaire. Guidelines from the Vietnam Ministry of Health and relevant references from the National Institute of Hygiene and Epidemiology were utilized develop this questionnaire (Vietnam Ministry of Health, 2009). This questionnaire was reviewed by five experts and pre-tested by five pregnant women and five child-bearing women within a year before actual implementation. 

The questionnaire comprised three parts: socio-demographic information and awareness and knowledge of cervical cancer and HPV vaccination. Awareness of cervical cancer and HPV vaccination was assessed by items concerning whether the interviewee had ever heard of cervical cancer or HPV vaccination, responding either yes or no. For those who had heard about either cervical cancer or HPV vaccination, detailed questions on cervical cancer (7 questions) or HPV vaccination (5 questions) were then asked with possible responses of true, false, unknown, or no response. Sometimes the correct answer to the question was “true,” and other times it was “false.” One point was given for each correct answer, and points were summed to create a summary knowledge score. If a question had more than one correct answer, 1 point was given for at least one correct answer. Higher knowledge scores indicated having greater knowledge of cervical cancer or HPV vaccination. All summary knowledge scores were divided into two groups (good and not good) according to mean scores described in a previous study (Prayudi et al., 2016). Beyond the mean scores, we further considered the distribution of knowledge scores (supplementary material) to identify an appropriate cut-off point (median value) for categorical decisions. Levels of knowledge of cervical cancer were categorized as “good” if more than five out of the seven questions were answered correctly or as “not good” if not. Levels of HPV knowledge were classified as “good” if more than two out of the five questions were answered correctly or as “not good” if not. Health status was self-reported by the participants using the scale of good, neutral, or not good.

All participants provided informed consent after explaining the survey’s objectives, that participation was voluntary, the confidentiality of this survey, the procedure, and potential benefits for the individual and communities. Only those who agreed to participate in this survey and provided consent were included in the study. The surveys were administered in either a health commune or a participant’s home upon request. Each participant took about 30-40 minutes to complete the interview and received a gift (50,000 VND) for their time spent.

*Ethical Review*

Ethical approval from the Institutional Ethical Review Board of the Institute for Preventive Medicine and Public Health, Hanoi Medical University in Hanoi, Vietnam (No 184/HMU IRB) was obtained for the study. Verbal informed consent was obtained from all participants. 

*Data analysis*

All completed questionnaires were entered into EpiData version 3.1 (EpiData Association, Denmark) independently by two people to ensure data accuracy. Descriptive analysis was conducted for socio-demographic factors and awareness and knowledge of cervical cancer and HPV vaccines. Pearson’s chi-square test and Fisher’s exact test were employed for bivariate analysis of all categorical variables, whereas an independent t-test was used to test differences in mean knowledge scores between women living in the two residential areas. All P values less than 0.05 were considered to be indicative of statistical significance. All statistical analyses were performed using STATA software, version 14 (Stata Corp. L.P., College Station, TX).

## Results

The demographical characteristics of the respondents are presented in Table 1. Of the 807 participants, about two-thirds (624 women) were younger than 30 years, and more younger women tended to reside in the rural area (Ba Vi) than in the urban area (Dong Da). Overall, women with less education and blue-collar workers or peasants more frequently resided in the rural area. While health insurance coverage almost doubled among women living in the urban area, about two-third self-reported as having good health regardless of their residential area. The number of women who answered that they had heard about cervical cancer was quite high (83.8%). Although the majority of respondents were aware of cervical cancer, more than one-fifth of women living in the rural area had never heard about it prior to this survey. Additionally, women living in the urban area were more likely to be aware of cervical cancer than women in the rural area (p<0.001). In contrast, nearly one-third of women answered that they had never heard about this vaccine before their participation in this study. In particular, more than 40.0% of women living in the rural area had not heard of HPV vaccination, whereas only 15% of women living in the urban area had never heard about it.

We assessed cervical cancer knowledge, after excluding 131 women who had never heard of cervical cancer prior to this study (Table 2). We found that over 40.0% of women did not know the answer or answered incorrectly for more than three out of seven questions. Specifically, there were many wrong answers to the following questions: “Cervical cancer is an inherited disease (right answer: false),” “Cervical cancer is caused by a virus (right answer: true),” or “Cervical cancer is an infectious disease (right answer: false).” Half did not know any signs of cervical cancer. Nevertheless, most of the participants knew that cervical cancer is preventable and that HPV vaccination is effective in preventing cervical cancer. Knowledge scores (ranging from 0-7 points) obtained by summing the number of correctly answered items in the survey are shown in Table 2. The average cervical cancer knowledge score was 4.60±1.43 out of a total possible score of 7 among the 676 participants who were aware of cervical cancer. Women living in rural areas showed statistically significantly higher knowledge scores than women living in urban ones.

Table 3 shows the participants’ knowledge of HPV vaccination among women who had ever heard of this vaccine. The majority of respondents showed very low levels of knowledge of HPV vaccination. More than 60.0% of respondents did know about the number of doses of HPV vaccine required in Vietnam, the ideal age for receiving the vaccine, or any side effects of HPV vaccination. Further, knowledge of HPV vaccination was significantly lower among women living in rural areas. Knowledge scores for HPV vaccination are shown in Table 3. The mean knowledge score for HPV vaccination was 1.53±1.35 out of a total possible score of 5. Women living in the urban area had significantly higher HPV knowledge scores than women living in the rural area.

**Table 1:** Demographic Characteristic

Variables	Overall		Urban		Rural		*P*-value
	n	%	n	%	n	%	
Total	807	100	400	49.6	407	50.4	
Age group							
<=25 years old	222	27.5	64	16	158	38.8	<0.001
26-30 years old	302	37.4	156	39	146	35.9	
>30 years old	283	35.1	180	45	103	25.3	
Marital status							
With spouse	801	99.3	398	99.5	403	99.0	0.43
Without spouse	6	0.7	2	0.5	4	1.0	
Child-birth status							
Having infants (0-1 years)	609	75.5	299	74.8	310	76.2	0.64
Pregnant	198	24.5	101	25.2	97	23.8	
Residential status							
Permanent residence	706	87.5	320	80	386	94.8	<0.001
Temporary residence	101	12.5	80	20	21	5.2	
Educational attainment							
College or higher	424	55.5	322	80.5	102	25.1	<0.001
High school or lower	383	47.5	78	19.5	305	74.9	
Occupation#							
White-collar	252	31.2	213	53.3	39	9.6	<0.001
Blue-collar/ Peasants	284	35.2	16	4	268	65.9	
Others	271	33.6	171	42.7	100	24.5	
Household monthly income per person*							
Moderate or higher	598	74.1	382	95.5	216	53.1	<0.001
Poor or near poor	209	25.9	18	4.5	191	46.9	
Health insurance card							
Yes	509	63.1	316	79	193	47.4	<0.001
No/No response	298	36.9	84	21	214	52.6	
Self-reported health status							
Good	502	62.2	255	63.8	247	60.7	<0.001
Neutral or not good	305	37.8	145	36.2	160	39.3	
Awareness of cervical cancer							
Yes	676	83.8	357	89.3	319	78.4	<0.001
No	131	16.2	43	10.7	88	21.6	
Awareness of HPV vaccination							
Yes	575	71.3	338	84.5	237	58.2	<0.001
No	232	28.7	62	15.5	170	41.8	

*Household income per person was categorized into either moderate if its income>=1,300,000VND or poor/near near-poor for the rest; # Blue collar/Peasants: sales, service and craft workers, skilled labors, machine operators, and peasants; White collar: managers, professionals, experts, engineers, and office workers; and Others: students, unemployed, and housewives

**Table 2:** Knowledge of Cervical Cancer by Residential Area among Women who were Aware of Cervical Cancer

Variables	Overall (n=676)		Urban (n=357)		Rural (n=319)		*P*-value
	n	%	n	%	n	%	
Cervical cancer is an inherited disease (False)							
True	171	25.3	101	28.3	70	21.9	0.16
False	388	57.4	195	54.6	193	60.5	
Unknown/ No response	117	17.3	61	17.1	56	17.6	
Cervical cancer is caused by a virus (True)							
True	213	31.5	147	41.2	66	20.7	<0.001
False	303	44.8	122	34.2	181	56.7	
Unknown/ No response	160	23.7	88	24.6	72	22.6	
Cervical cancer is an infectious disease (False)							
True	134	19.8	72	20.2	62	19.4	0.83
False	442	65.4	235	65.8	207	64.9	
Unknown/ No response	100	14.8	50	14	50	15.7	
Population at high cervical cancer risk							
Women ever given birth	512	75.7	258	72.3	254	79.6	0.03
Older adults	21	3.1	16	4.5	5	1.6	0.03
Unfaithful people	106	15.7	54	15.1	52	16.3	0.68
Women with poor (gynecological)	153	22.6	53	14.9	100	31.4	<0.001
hygiene behavior							
Point out at least one correct	566	83.6	292	81.8	273	85.6	0.19
Unknown/ No response	111	16.4	65	18.2	46	14.4	
Signs of cervical cancer							
Abnormal vaginal bleeding	227	33.6	75	21	152	47.7	<0.001
Unusual discharge from vagina	175	25.9	66	18.5	109	34.2	<0.001
Urinary incontinence	64	9.5	10	2.8	54	16.9	<0.001
Bleeding after intercourse	153	22.6	29	8.1	124	38.9	<0.001
Pain during sexual intercourse	118	17.5	36	10.1	82	25.7	<0.001
Back pain	52	7.7	8	2.2	44	13.8	<0.001
Point out at least one sign	331	49	122	34.2	209	65.5	<0.001
Unknown/ No response	345	51	235	65.8	110	34.5	
Cervical cancer can be prevented (True)							
True	618	91.4	323	90.5	295	92.5	0.15
False	14	2.1	11	3.1	3	0.9	
Unknown/ No response	44	6.5	23	6.4	21	6.6	
HPV vaccine is one of the most effective ways to prevent cervical cancer * (True)							
True	554	89.6	295	91.3	259	87.8	0.32
False	51	8.3	23	7.1	28	9.5	
Unknown/ No response	13	2.1	5	1.6	8	2.7	
Summary Score							
Average score (mean, SD)	4.6	1.43	4.51	1.46	4.71	1.39	0.07
Good knowledge (n,%)(>=5/7 right answers)	402	59.5	197	55.2	205	64.3	0.02
Not good (n,%) (<5/7 right answers)	274	40.5	160	44.8	114	35.7	

*, Among women who said that cervical cancer can be prevented; SD, standard deviation

**Table 3:** Knowledge of HPV Vaccination According to Residential Area among Women Aware of This Vaccine

Variables	Overall (n=575)		Urban (n=338)		Rural (n=237)		*P-* value
	n	%	n	%	n	%	
HPV vaccination comprises three doses in Vietnam							
True	96	16.7	91	26.9	5	2.1	<0.001
False	143	24.9	83	24.6	60	25.3	
Unknown/No response	336	58.4	164	48.5	172	72.6	
HPV vaccines should be given at the ages of 9-26 years							
True	218	37.9	129	38.2	89	37.6	<0.001
False	159	27.7	113	33.4	46	19.4	
Unknown/No response	198	34.4	96	28.4	102	43.0	
Women who have been sexually active can receive HPV vaccination							
True	263	45.7	154	45.6	109	46.0	<0.001
False	160	27.8	112	33.1	48	20.3	
Unknown/No response	152	26.5	72	21.3	80	33.7	
Presence of HPV should be tested before receiving vaccination for sexually active women*							
True	210	79.9	126	81.8	84	77.1	0.13
False	34	12.9	21	13.6	13	11.9	
Unknown/No response	19	7.2	7	4.6	12	11.0	
Side effect of HPV vaccines							
Point out at least one side effect	95	16.5	59	17.5	36	15.2	0.001
No side effects	98	17.0	73	21.6	25	10.6	
Unknown/No response	382	66.5	206	60.9	176	74.2	
Summary Score							
Average score (mean, SD)	1.53	1.35	1.65	1.37	1.36	1.3	0.01
Good knowledge(n,%) (>=2/5 right answers)	286	49.7	180	53.3	106	44.7	0.04
Not good (n,%) (<2/5 right answers)	289	50.3	158	46.7	131	55.3	

*, Among women who said that sexually active women can receive the HPV vaccine

## Discussion

This is the first study to quantitatively investigate knowledge of cervical cancer and HPV vaccination among women of child-bearing age in Vietnam, where cervical cancer remains a major public health concern. This study highlights limited understanding of cervical cancer and HPV among these women who are primarily responsible for caring for their families. Therefore, tailored interventions are needed to increase public awareness and knowledge of cervical cancer and its vaccination.

As expected, the majority of respondents answered that they had heard about cervical cancer and HPV vaccination. More women said that they had heard about cervical cancer than the HPV vaccine. Similarly, previous studies have also reported greater awareness of cervical cancer than vaccination of HPV in other countries and ethnic groups (Bodson et al., 2016; Borlu et al., 2016; Saqer et al., 2017; Islam et al., 2018). This can be explained by the fact that cervical cancer is currently a great concern in many countries; however, HPV vaccines are not often covered in national immunization programs. In Vietnam, a government program implemented in 2008 was designed to cover all aspects required for the application of an HPV vaccination program; however, even though the feasibility of HPV vaccination delivery was proven, a national program for HPV vaccination in Vietnam has yet to be fully implemented. This may account for the poor awareness and knowledge of HPV vaccination among Vietnamese women. 

Regarding knowledge of cervical cancer, scores for correct answers to a total of seven questions were recorded in the current study, with a mean of 4.60 ±1.43 questions answered correctly. This mean score is much higher than reported in studies in other countries (Kietpeerakool et al., 2009; Wong and Sam, 2010; Rashwan et al., 2011; Wong, 2011; Zhao et al., 2012; Poole et al., 2013; Fernandez et al., 2014; Perlman et al., 2014; Strohl et al., 2015). Recently, with greater coverage from broadcasting programs, as well as the internet, the spread of information has become relatively easier to achieve than ever. Further, women of reproductive age might have a better knowledge of cervical cancer, as they may be more concerned with reproductive health.

Concerning HPV vaccination, the majority of respondents lacked knowledge thereof, with a mean of 1.53 (SD=1.35) questions answered correctly for a total of five questions. Similarly, low mean scores have often been reported elsewhere (Do et al., 2009; Halliday et al., 2013; Fernandez et al., 2014; Kruiroongroj et al., 2014; Perlman et al., 2014; Koc and Cinarli, 2015; Strohl et al., 2015; Bodson et al., 2016; Yu et al., 2016; Turhan et al., 2019). Furthermore, in the current study, knowledge scores for HPV vaccination were markedly lower among women living in a rural area than among those residing in an urban area. Interestingly, while the women residing in an urban area showed relatively greater HPV vaccine knowledge, they had a lower understanding of cervical cancer, compared with women who lived in a rural area. There may be several possible explanations for this: The first explanation may be related to differences in education and health promotion programs in each area. In urban areas, where various prevention services are present, education and health promotion programs for cervical cancer typically focus on HPV vaccination, whereas, in areas where prevention programs are not well organized, educational and promotional materials mainly concentrate on features and risk factors for the disease. Another explanation may be that greater cervical cancer knowledge, which was noted for women living in a rural area, might be associated with higher incidence rates of cervical cancer in rural areas (Baker et al., 2000; Benard et al., 2008). 

Taking these findings together, we suggest that a comprehensive educational campaign on HPV vaccination that describes the number of required HPV vaccine shots, recommended ages for vaccination, and possible side effects is needed. Initial efforts that enable future education programs ought to be built on existing knowledge, and simultaneous attempts to address common misconceptions or unknown factors could be beneficial. Trusted persons, such as health workers or teachers, as well as influential individuals, may prove effective in disseminating such information. Meanwhile, education programs should seek to take advantage of communal health centers in order to foster community awareness, and distributing information via pamphlets or leaflets at local health centers in the countryside has been recommended as a means of increasing the acceptability of HPV vaccination in other countries (Ali, 2017).

Some limitations to this study warrant consideration. First, our data were employed in a cross-sectional study, and thus, it is beyond the present scope to determine any causal relationships. Second, the study results do not represent the entire Vietnamese population, because we targeted only two regions in this study. Lastly, this study did not include women of younger age (e.g., 12 years of age), which is the target age for receiving HPV vaccination. Additionally, the study population of pregnant women and women who recently gave birth do not represent the entire population of Vietnamese women. 

Despite these limitations, our study has several strengths. First, this is the first study to investigate the associations between socio-demographic factors and levels of knowledge on cervical cancer and the HPV vaccine among Vietnamese women. Our findings indicate a need for public health policymakers to push forward step-by-step strategies for cervical cancer prevention in the near future. Second, women of child-bearing age, who are primary caretakers of children and protectors of the health for each family, were recruited, and we assessed their awareness and levels of knowledge on cervical cancer and HPV vaccine, as their knowledge about cervical cancer and HPV vaccination can strongly affect their intentions to receive vaccination themselves and for their daughters.

In conclusion, despite high levels of awareness, knowledge on cervical cancer and HPV vaccination was

lacking among women of child-bearing age in both urban and rural areas of Hanoi, Vietnam. Our findings underscore a need to develop well-designed educational programs on cervical cancer and HPV vaccination in Vietnam and other countries.
